# Increased Cervical Disc Height and Decreased Neck Pain and Disability Following Improvement in Cervical Lordosis and Posture Using Chiropractic BioPhysics

**DOI:** 10.3390/bioengineering13020229

**Published:** 2026-02-15

**Authors:** Evan A. Katz, Seana B. Katz, Sophie F. Katz, Curtis A. Fedorchuk, Cole G. Fedorchuk, Douglas F. Lightstone

**Affiliations:** 1 Private Practice, Boulder, CO 80302, USA; evan@katzchiropractic.com (E.A.K.);; 2 Institute for Spinal Health and Performance, Cumming, GA 30041, USA

**Keywords:** Chiropractic BioPhysics, degenerative disc disease, cervical disc height, cervical lordosis, anterior head posture, cervical spine, posture, disc height, cervical spine morphology, spine rehabilitation

## Abstract

Background/Objectives: Cervical degenerative disc disease (DDD) is associated with decreased disc height, spinal arthrosis, decreased spinal stability, neck pain (NP), and increased years living with disability and global disease burden. Methods: A total of 64 patients (19 males, 45 females) between 23 and 77 years (mean age of 49.05 ± 3.34 years) presented to a private practice with NP and disability. Pre-treatment radiographs revealed decreased cervical curvature (ARA C2–C7) measuring −6.18 ± 3.06° (ideal is −42.0°), anterior head translation (Tz C2–C7) measuring 22.03 ± 2.39 mm (ideal is 0 mm), anterior cervical disc height (ADH C2–C7) measuring 3.68 ± 0.20 mm, and posterior cervical disc height (PDH C2–C7) measuring 3.21 ± 0.15 mm. Pre-treatment NP numeric rating scale (NRS) scored 6.66 ± 0.27, and neck disability index (NDI) scored 40.28 ± 1.42%, indicating moderate disability due to NP. Patients were treated using Chiropractic BioPhysics^®^ (CBP^®^) Mirror Image^®^ spinal rehabilitation for mean values of 37.80 ± 2.44 treatment visits over 19.48 ± 3.89 weeks at a frequency of 2.89 ± 0.45 treatment visits per week. Results: Post-treatment radiographs revealed improvements in ARA C2–C7 to −19.95 ± 3.05°, Tz C2–C7 to 12.11 ± 2.34 mm, ADH C2-C7 to 5.19 ± 0.21 mm, and PDH C2-C7 to 4.36 ± 0.16 mm. Post-treatment patient-reported outcomes showed improvements in NP NRS to 1.52 ± 0.26 and NDI to 12.66 ± 0.96, indicating minimal NP and disability. Conclusions: CBP^®^ helps improve sagittal cervical spinal alignment and posture, which may help improve cervical disc height and NP and disability in adult patients with cervical DDD.

## 1. Introduction

Neck pain (NP) is one of the most common conditions causing significant pain, disability, and financial burden is NP. It not only accounts for significant personal hardship, but also has an impact on families, the healthcare system, and national economies [1]. Among 154 conditions, NP accounted for the largest portion of US health care spending in 2016, estimated at USD 134.5 billion. Globally, in 2019, there were 222.7 million prevalent cases, 47.5 million incident cases, and 22.1 million years lived with disability (YLD) cases, and the number of prevalent cases, incident cases, and years lived with disability is increasing [2].

Degeneration of the cervical intervertebral disc (IVD) is a frequent source of NP [3], which results from abnormal cervical structure and function [3]. In addition to NP, cervical disc degeneration symptoms include neck and upper back stiffness, reduced cervical range of motion (ROM), headache (HA), shoulder pain, radicular arm pain, ocular and vestibular dysfunction, and anterior chest wall pain [3]. Increased levels of inflammatory cytokines, which are released by the disc cells themselves and encourage matrix damage, are indicative of disc degeneration [3]. Inflammation triggers a pathogenic cascade leading to accelerated aging, apoptosis of the IVD, as well as nerve and blood vessel ingrowth, causing further pain, dysfunction, and degenerative changes [3].

Unhealthy changes in cervical spinal alignment and posture have been shown to cause cervical spine degeneration, leading to degenerative disc disease (DDD). Loss or reversal in cervical lordosis [4,5,6,7,8,9], increased anterior head posture [10,11], and abnormal cervical biomechanics [12,13,14,15] are shown to have a causative effect. This begs the question of whether improving cervical spinal alignment and posture can have a positive impact on cervical disc degeneration measures. Other published literature has focused on the effects of spinal therapies on the functional, symptomatic, and subjective experience of patients with cervical disc degeneration.

The objective of this case series is to report on improved functional, symptomatic, and radiographic findings consistent with cervical disc degeneration in 64 patients following structural cervical spine rehabilitation.

## 2. Materials and Methods

### 2.1. Patients

This retrospective consecutive case series involved a records review of patients and documenting the clinical results of all patients who met the inclusion/exclusion criteria. There is no control group. This study was confirmed for institutional review board (IRB) exemption by Advarra IRB, registered with OHRP and FDA under IRB#00000971 on 26 February 2025, according to Exemption Category 4: secondary research of identifiable private information for which consent is not required due to information recorded in such a manner that the identity of the human subjects cannot readily be ascertained directly or through identifiers linker to subjects, the investigator will not contact the subjects, and the investigator will not re-identify subjects. Informed consent was waived because of the retrospective nature of the study. Patients were selected from a records review from a chiropractic clinic with advanced training in structural rehabilitation of the spine and that follows Chiropractic BioPhysics^®^ (CBP^®^) protocols [12]. The patients selected for this case series met certain inclusion criteria:

1. Physical examination revealed neck pain (International Classification of Diseases, Tenth Revision (ICD-10) M54.2), abnormal cervical posture (ICD-10 R29.3), reduced neck mobility (ICD-10 Z74.09), and cervical spine dysfunction (ICD-10 M99.01).
2. Health history revealed the patients reported that they sought medical treatment, traditional chiropractic adjustments, and(or) physical therapy (PT) previously and that their symptoms had not improved.
3. Patient-reported outcomes (PRO) using the NP numeric rating scale (NRS) and neck disability index (NDI) revealed moderate-to-severe NP and disability.
4. NLC radiographs including C2 to C7 revealed cervical hypolordosis (ICD-10 M95.3), degeneration of the cervical spine (ICD-10 M47.892), and cervical disc degeneration (ICD-10 M50.30).
5. Compliance with treatment recommendations, including CBP^®^ spinal rehabilitation, Mirror Image^®^ (MI) chiropractic adjustments, therapeutic spinal exercises, and mechanical spinal traction, followed by post-treatment neutral lateral cervical (NLC) radiographs for comparison to pre-treatment radiographs.

Patients were not selected for this case series if they met certain exclusion criteria:

1. Presence of red flags or contraindications for chiropractic adjustments, therapeutic spinal exercises, or mechanical spinal traction to the cervical spine.
2. Presence of cervical or cervicothoracic scoliosis or lateral translations of C2 with respect to T4 (Tx C2–C4) measuring 7 mm or greater [13].

All methods were carried out according to relevant guidelines and regulations. This case series is a retrospective analysis of patients treated in a private practice according to standards of care. This case series did not involve any experimentation on human or animal subjects, and all data and identifying content have been anonymized.

### 2.2. Patient Reported Outcomes

PROs provide critical information regarding patients’ symptoms and conditions. PROs may provide qualitative (e.g., mild, moderate, severe, etc.) and(or) quantitative (e.g., scores, percentages, etc. of 0–10, 0–100, etc.) measures. Patient-reported outcome measures (PROM) are the tools or instruments used to measure PROs, including functional status, symptom(s), and symptom burden for health conditions [14].

#### 2.2.1. Neck Pain Numeric Rating Scale

An NP NRS is a PROM that allows patients to quantify the severity of their NP on a scale from 0 to 10, where 0 represents no NP, and 10 represents the maximum severity of NP. NP NRS was administered at pre-treatment and post-treatment exams. The minimally clinically important difference (MCID) needs to be −1.3 points on the NRS to represent a significant improvement in pain [15].

#### 2.2.2. Neck Disability Index

NDI assesses the impact of NP on activities of daily living (ADL) and provides a quality and quantity of disability due to NP on a scale of 0 to 100, where 0 is no disability due to NP and 100 is complete disability due to NP [16]. NDI was administered at pre-treatment and post-treatment exams. NDI changes of 21% are considered clinically meaningful [17].

### 2.3. Radiographic Analysis

**Figure 1:**
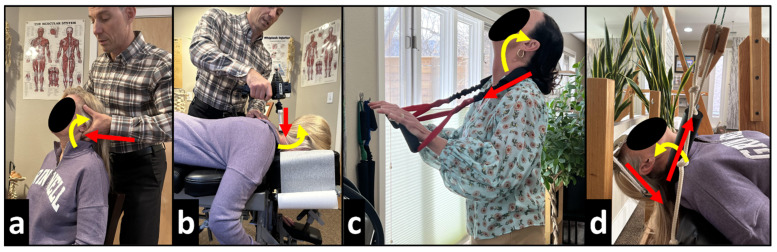
(**a**–**d**) The red arrow indicates the vector of corrective force (in this figure, the force is the MI corrective adjustment) and the yellow arrow indicates the MI corrective positioning of the patient to assist the adjustment. (**a**) Seated corrective adjustment involving a P-A manual thrust with the neck positioned into extension (MI); (**b**) Prone corrective adjustment on an adjusting table involving P-A instrument-assisted thrust with the neck positioned into extension (MI); (**c**) The patient is performing cervical extension repetitions while providing a P-A counterstress at the mid-neck while standing on a WBV platform; (**d**) The patient is performing cervical compression-extension traction with a P-A counterstress at the mid-neck.

**Figure 2:**
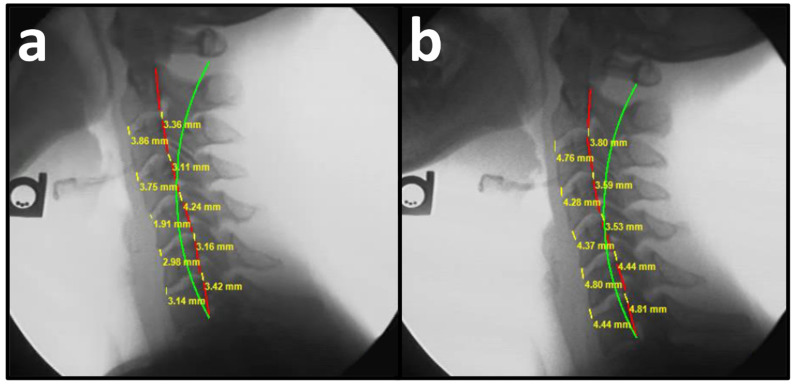
(**a**,**b**) Pre-treatment and post-treatment neutral lateral cervical (NLC) radiographs of a 29-year-old female initially reporting 7/10 neck pain (NP) and 40% (moderate) disability due to NP. The green line represents a normal, ideal sagittal cervical alignment, the red line represents the actual posterior tangent lines of the C2 to C7 vertebrae, and the yellow lines and numbers represent the ADH C2–C7 and PDH C2–C7. (**a**) Pre-CBP treatment NLC radiograph with an ARA C2–C7 measuring −3.40° (ideal is −42°), Tz C2–C7 measuring 24.10 mm, ADH C2–C7 measuring 3.13 mm, PDH C2–C7 measuring 3.46, and a 2nd order sagittal buckling with cervical kyphosis at C5–C7; (**b**) Post-CBP treatment NLC radiograph after 31 visits over 10 weeks with improvements in ARA C2–C7 to −19.00°, Tz L5-S1 to 15.10 mm, ADH C2–C7 to 4.53 mm, PDH C2–C7 to 4.03, and reduction in cervical kyphosis at C5–C7.

Spinal radiographs are necessary to assess for red flags or contraindications to spinal treatment, pathology, spinal alignment, and vertebral subluxations as a means to determine correct approaches to structural spinal rehabilitation, as well as patient progress in a spinal correction plan [18]. The Harrison posterior tangent method (HPTM) provides measurements of regional and intersegmental vertebral angles and regional and intersegmental vertebral translations. On NLC radiographs, cervical angles are measured by drawing a line tangent to the posterior margin of each vertebral body from C2 to C7. Measurements of a spinal region provide the absolute rotation angle (ARA). Anterior disc height (ADH) on a sagittal radiograph is measured from the anterior-inferior aspect of the superior vertebra and the anterior-superior aspect of the inferior vertebra. Posterior disc height (PDH) on a sagittal radiograph is measured from the posterior-inferior aspect of the superior vertebra and the posterior-superior aspect of the inferior vertebra (Figure 1 and Figure 2). These methods have been shown to be valid and reliable [19]. The measurement error of vertebral height amounts to 3.9%. The error of disc height amounts to 5.7% (0.34 mm with a mean vertebral depth of 17 mm) [19]. The relative measurement error in vertebral height amounts to 2.2%; for a vertebra of 30 mm height, this corresponds to an error of approximately 0.7 mm. The error in sagittal plane displacement amounts to 0.015 (measured in units of mean vertebral depth); for a vertebra of 35 mm depth, this corresponds to an error of 0.5 mm. The relative error in disc height amounts to 4.15%; for a disc of 10 mm height, this corresponds to an error of approximately 0.42 mm [20]. Intersegmental translation measurements have shown mean absolute deviation (MAD) of 0.23–0.46 mm for interexaminer reliability and 0.14–0.21 mm for intraexaminer reliability [21]. Sagittal vertical axis (C2-C7) has shown an MAD of 0.3 mm [22].

Pre-CBP^®^ treatment radiographs were taken at initial examination prior to any treatment intervention(s). Post-CBP^®^ treatment radiographs were taken at least 24 h after completion of treatment. This approach is employed to rule out short-term effects of spinal rehabilitation on spinal alignment and posture. Patient positioning was consistent with procedures taught in CBP^®^ technique, which are very reliable and repeatable [23]:

For lateral cervical radiographs, the patient’s shoulders were positioned perpendicular to the radiographic bucky, and the patient was instructed to close his/her eyes, to flex and extend the head twice, and come to a resting neutral position. This neutral resting posture is that in which the patient perceives his/her head to be looking straight forward. The patient then opens his/her eyes and is instructed to look straight ahead without moving. The patient’s abnormal sagittal plane posture is left as is (i.e., it is not guided toward an ideal neutral position) [24].

The lateral cervical is taken at the tube distance of 101.6 cm (40 inches), with the central ray located approximately at the C4 level.

The spinal postural and radiographic analysis uses a right-hand, thumb-up Cartesian coordinate system to illustrate translations and rotations in and around x, y, and z-axes of the head, thorax, and pelvis [25]. A positive or negative sign indicates direction of translation in or rotation around the x, y, and z-axes; the first letter indicates translation (T) or rotation (R); the second letter indicates the axis; the alphanumeric after the anatomical landmarks involved (head (H), thorax (T), pelvis (P) or vertebrae) [12].

NLC radiographs were analyzed using PostureRay Electronic Health Records (EHR) Software (PostureCo, Inc., Trinity, FL, USA) per the Harrison posterior tangent method for spine views in the sagittal plane [23,26,27] at a CBP spinal rehabilitation clinic by a doctor with advanced training in CBP methods and CBP instructor status with over 20 years of clinical and radiography experience. The examiner was blinded to the radiographic measurements. Unmarked, anonymized radiographs were provided at random to be analyzed. The examiner was unaware of the patient or whether the radiograph was a pre- or post-treatment radiograph. These examination and analysis methods are valid [24,25,28,29,30], reliable, and repeatable [23,24,25,26,27,31], as is posture [24].

### 2.4. Interventions and Outcomes

The treatment visits for the patients consisted of MI corrective adjustments, therapeutic spinal exercises, and mechanical spinal traction per CBP^®^ protocols [12] administered at a CBP spinal rehabilitation clinic by a doctor with advanced training in CBP methods and CBP instructor status with over 20 years of experience. CBP focuses on normalizing spinal alignment and biomechanics by applying MI exercises, adjustments, and traction to address musculature, neurology, and connective tissue, respectively [12].

MI corrective adjustments were delivered while seated or prone using an OMNI elevation adjusting table with sectional drop-mechanisms and setting up the patient in the position obtained during MI position, followed by a posterior-to-anterior (P-A) thrust to the mid-lower cervical vertebrae by hand (Figure 1a) and also using the IMPAC Pro-ArthroStim Instrument (I.M.P.A.C. Inc., Salem, OR, USA) (Figure 1b). MI adjusting stimulates mechanoreceptors and proprioceptors to retrain the body’s central nervous system (CNS) to adapt to normal, healthy posture [12].

The MI therapeutic spinal exercises involved the patient performing cervical extension repetitions while providing a posterior-to-anterior P-A counterstress at the mid-neck using the CBP Pro-Lordotic Neck Exerciser (Chiropractic BioPhysics, Eagle, ID, USA) on a whole-body vibration (WBV) Power Plate (Power Plate Performance Health Systems, LLC, Northbrook, IL, USA) (Figure 1c). The patient held the final position for 10 s before relaxing and repeating for 50 repetitions. MI exercises strengthen weak musculature and lengthen tight musculature that have adapted to unhealthy posture to correct and maintain corrections in spinal alignment and postural abnormalities [12].

MI mechanical spinal traction was performed using a DeGeorge Regainer Compression Extension Traction unit (Promote Chiropractic, Inc., Saugus, MA, USA) with a Total Target Force Counterstress Traction unit (Promote Chiropractic, Inc., Saugus, MA, USA). A force combining cervical spine extension and compression was applied using a fulcrum behind the neck that could be pulled P-A and a forehead harness that could increase cervical extension but also pull caudally to induce compression. MI traction was performed for periods starting at 3 min and increasing by 2 min with each visit until 15 min was reached (Figure 1d). MI traction intensity was set to patient tolerance on the day of traction. As cervical spine extension was increased with the P-A pull at the cervical spine, patients were instructed to communicate when they felt the intensity was too much to tolerate, at which point the P-A pull intensity was decreased to provide the strongest pull within patient tolerance. MI traction allows for viscoelastic plastic deformation of spinal ligaments [12] and corrects the patient’s abnormal posture by initiating muscle and ligament creep, creating permanent restorative change.

Any number of variables can affect the threshold of patient tolerance to MI corrective adjustments, therapeutic spinal exercises, and mechanical traction in general, or even at a given point in time (e.g., physical, psychological, emotional states or symptoms). It is up to the practitioner to work with the patient to help navigate obstacles these variables may pose in maximizing the benefits of MI traction (or any other treatments or therapies).

### 2.5. Statistical Analyses

A total of 64 patients were included in the analytic sample. The primary outcomes were ARA C2–C7 and Tz C2–C7. The secondary outcomes were ADH C2–C7, PDH C2–C7, NP NRS, and NDI. Before pooling the data, patient baseline characteristics were summarized, including sex, height, weight, age, number of treatment visits, and duration of treatment.

A correlation analysis was conducted for all the outcome variables to determine if one variable changed in value, would the other variable tend to change in a specific direction [32]. Understanding that relationship was useful since highly correlated variables would not be recommended for being put into multivariable analyses. The correlation coefficient was a quantitative assessment that measured both the direction and the strength of the tendency to vary together. Pearson’s correlation coefficient was used to assess the analysis.

A pre–post analysis was conducted to check the average effect of the independent variables on the outcomes and identified how much the effect varied by the level of significance [33], through linear regression models. Multivariable analyses were used to control the effect of each variable. The analysis identified whether the effect varied for each variable by checking the level of significance with the following equation:

(1) $$DV=\;\beta_{0}+\beta_{1}Post\;Treatment+\sum_{i=2}^{n}{\beta_{i}IV}_{i}+\;\varepsilon_{i}$$

The method captured the effect on the outcome from each independent variable. Model components included the dependent variable (DV), the independent variables (IV_i_), and the idiosyncratic error term (ε_i_). Given the patient with specific characteristics and conditions, the study would expect to see the predicted outcomes (primary and secondary outcomes) adjusted by different confounders. Therefore, β_i_ captured the average effect from the different factors on this prediction, determining whether the variable effect would reduce or increase the prediction of outcomes. In addition, the strength of the effect from each of the variables would be evaluated through the statistical test significance, determining the *p*-value at 95% confidence interval [34].

Furthermore, the post hoc power analyses were conducted based on the output for primary outcomes [35]. A post hoc power analysis can provide insight into the adequacy of the sample size used in the study, though it cannot change the results or conclusions.

All the summaries and statistical analyses were performed using the current version of the open-source statistical programming language SAS 9.4. Summary statistics used PROC FREQ/PROC MEANS, *t*-test used PROC TTEST, descriptive statistics used PROC ANOVA, correlation analysis used PROC CORR, and the multivariable linear model for pre–post analysis used PROC GLM.

## 3. Results

Patient baseline characteristics and demographics are presented in Table 1. This case series reports on 64 patients (45 females and 19 males) with a mean age of 49.05 ± 3.34 years, height of 171.93 ± 1.85 cm, and mass of 68.12 ± 2.53 kg (Table 1) who presented with a chief complaint of NP with associated upper trapezii pain, neck stiffness, and reduced cervical ROM. The patients reported limitations in performing ADL. The patients reported that they sought medical treatment, traditional chiropractic adjustments, and(or) PT prior to corrective chiropractic spinal rehabilitation and that their symptoms had not improved.

### 3.1. Pretreatment

**Figure 3:**
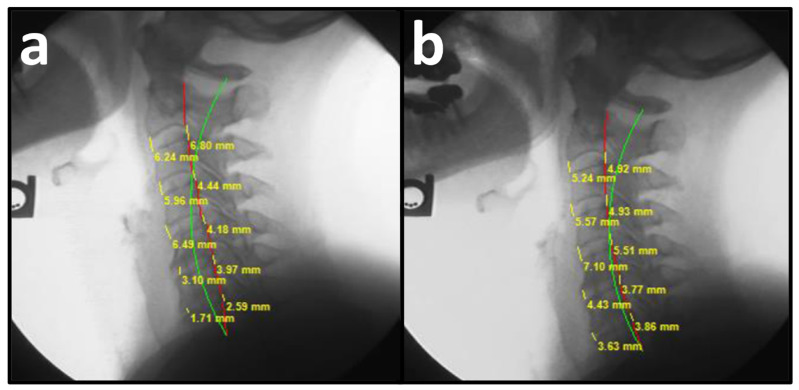
(**a**,**b**) Pre-treatment and post-treatment neutral lateral cervical (NLC) radiographs of a 72-year-old female initially reporting 7/10 neck pain (NP) and 38% (moderate) disability due to NP. The green line represents a normal, ideal sagittal cervical alignment, the red line represents the actual posterior tangent lines of the C2 to C7 vertebrae, and the yellow lines and numbers represent the ADH C2–C7 and PDH C2–C7. (**a**) Pre-CBP treatment NLC radiograph with an ARA C2–C7 measuring −3.80° (ideal is −42°), Tz C2–C7 measuring 20.00 mm, ADH C2–C7 measuring 4.70 mm, PDH C2–C7 measuring 4.40, and a 2nd order sagittal buckling with cervical kyphosis at C5–C7; (**b**) Post-CBP treatment NLC radiograph after 32 visits over 17 weeks with improvements in ARA C2–C7 to −23.20°, Tz L5–S1 to 14.40 mm, ADH C2–C7 to 5.20 mm, PDH C2–C7 to 4.60, and reversal of cervical kyphosis at C5–C7.

PRO revealed a mean NP NRS score of 6.66 ± 0.27, indicating moderate NP, and a mean neck disability index NDI score of 40.28 ± 1.42%, indicating moderate disability due to NP. Pretreatment NLC radiography revealed a mean translation in the sagittal plane (*z*-axis) of C2 with respect to C7 vertebrae (Tz C2–C7) measuring 22.03 ± 2.39 mm (ideal is 0 mm and average is 15 mm), mean cervical lordosis (absolute rotational angle) from C2 with respect to C7 vertebra (ARA C2–C7) measuring −6.18 ± 3.06° (normal is −42° and average is 34°), disc height of the anterior aspect of C2 to C7 vertebrae measured from the anterior-inferior of the superior vertebra to the anterior-superior aspect of the inferior vertebra (ADH C2–C7) measuring 3.68 ± 0.20 mm, and disc height of the posterior aspect of C2 to C7 vertebrae measured from the posterior-inferior of the superior vertebra to the posterior-superior aspect of the inferior vertebra (PDH C2–C7) measuring 3.22 ± 0.15 mm (Figure 2a and Figure 3a, Table 2) [34].

### 3.2. Post-Treatment

After a mean of 37.80 ± 2.44 treatment visits over 19.53 ± 3.89 weeks at a rate of 2.87 ± 0.44 treatment visits per week, the patients reported improvements in NP NRS to 1.52 ± 0.26 (indicating minimal NP) and NDI to 12.66 ± 0.96% (indicating minimal disability due to NP). Post-treatment NLC radiographs revealed improvements in mean Tz C2–C7 to 12.11 ± 2.34 mm, mean ARA C2–C7 to −19.95 ± 3.05°, mean ADH C2–C7 to 5.19 ± 0.21 mm, and mean PDH to 4.35 ± 0.16 mm (Figure 2b and Figure 3b, Table 2).

The changes in pre- to post-treatment ADH and PDH measurements are statistically significant. Based on pre-CBP treatment mean ADH and PDH C2–C7 measurements of 3.68 mm and 3.21 mm, 5.7% error [19] would be 0.21 mm and 0.18 mm. Based on post-CBP treatment mean ADH and PDH C2–C7 measurements of 5.19 mm and 4.36 mm, 5.7% error would be 0.30 mm and 0.25 mm. These numbers support the conclusion that these changes are also clinically significant. Further, mechanical alterations of the disc as much as 1 mm of change in disc height can significantly affect mechanical and inflammatory responses within the disc tissue, which influence nociceptive pathways and pain generation [36].

### 3.3. Statistical Analyses

**Figure 4:**
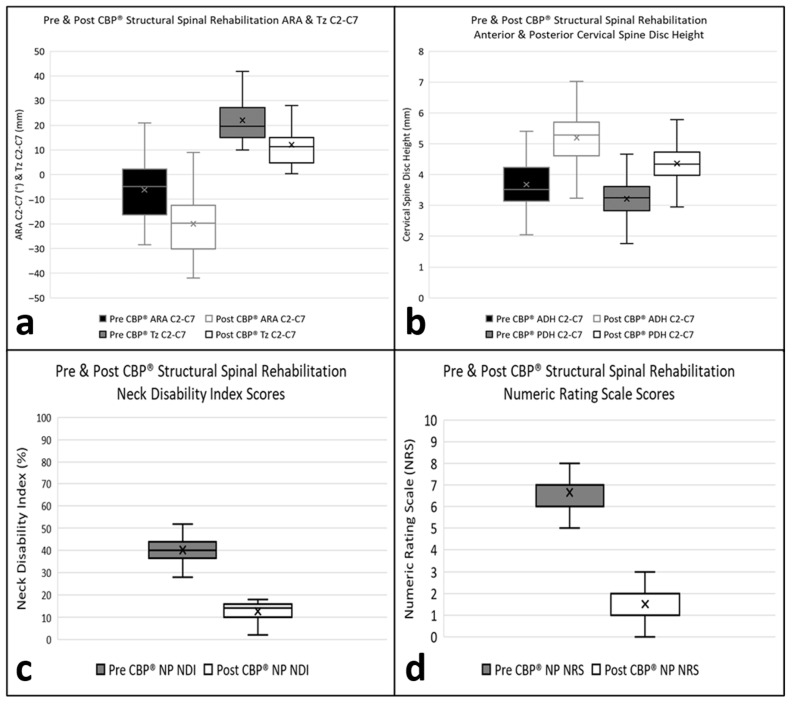
(**a**–**d**) Distribution of primary (ARA C2–C7 and Tz C2–C7) and secondary (NP NRS and NDI) outcomes of pre- and post-CBP structural spinal rehabilitation through box plots. (**a**) Cervical lordosis and anterior head translation improved from pre- to post-CBP structural spinal rehabilitation; (**b**) Anterior and posterior cervical disc heights improved from pre- to post-CBP structural spinal rehabilitation; (**c**,**d**) NP and disability due to NP improved from pre- to post-CBP structural spinal rehabilitation.

The primary and secondary outcomes were compared between pre-treatment and post-treatment with *t*-tests. The mean difference with 95% confidence interval, as well as *p*-value, was calculated (Table 2). Based on the descriptive statistics, the post-treatment groups yielded significantly different outcomes compared to the pre-treatment groups, as *p*-values were all less than 0.001. Note that these differences were demonstrated through box plots (Figure 4a–d).

**Figure 5:**
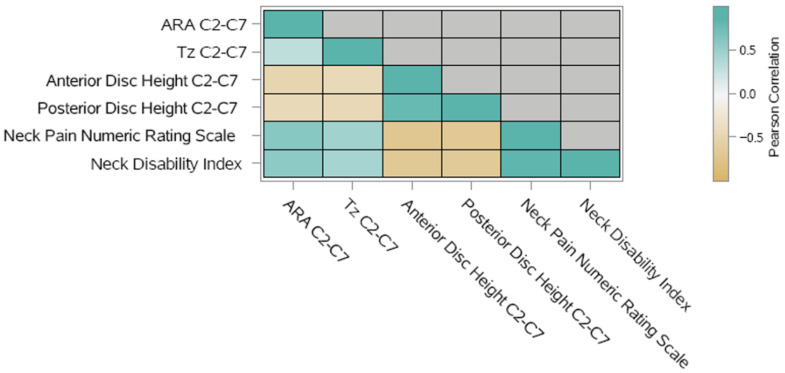
Heat map of Pearson’s correlation coefficient analyses of primary and secondary outcomes. The darker color (either green or tan) indicated a higher correlation to positive or negative.

A correlation analysis was conducted for all the outcomes to determine if one variable changed in value, would the other variable(s) tend to change in a specific direction. Understanding that relationship was useful since highly correlated variables would not be recommended for being put into the multivariable regression model. The correlation coefficient was a quantitative assessment that measured both the direction and the strength of the tendency to vary together. Pearson’s correlation coefficient was used to assess the analysis [37]. Based on the results of the correlation analysis, the heatmap was plotted (Figure 5). Note that the darker color (green or brown) indicates a higher correlation to positive or negative. The blocks on the diagonal represented the correlation with the variable itself (CORR = 1), which would not be considered in the analysis. From the output of correlation, it would be concluded that the positive associations were between Tz C2–C7 and ARA C2–C7, NP NRS and ARA C2–C7, NP NRS and Tz C2–C7, NDI and ARA C2–C7, NDI and Tz C2–C7, and NP NRS and NDI; in other words, as cervical lordosis and anterior head translation improved, so too did NP and disability. The negative associations were between ADH C2–C7 and ARA C2–C7, PDH C2–C7 and ARA C2–C7, ADH C2–C7 and Tz C2–C7, PDH C2–C7 and Tz C2–C7, NP NRS and ADH C2–C7, NP NRS and PDH C2–C7, NDI and ADH C2–C7, and NDI and PDH C2–C7; in other words, as cervical lordosis and anterior head translation improved, so too did anterior and posterior cervical disc height. And as anterior and posterior disc height improved, so too did neck pain and disability.

The full output of multivariable linear regression on the primary outcomes comparing pre- and post-treatment was calculated (Table 3). The results show that with the adjustments of ADH C2–C7, PDH C2–C7, NP NRS, and NDI, ARA C2–C7 increased 37.61 units on average for post-treatment compared to post-treatment with statistical significance of a *p*-value less than 0.05, but Tz C2–C7 was not statistically significant after the adjustment, with a *p*-value above 0.05.

In the study, we evaluated two PROs (NP NRS and NDI) and four radiographic outcomes (Mean ARA C2–C7, Mean Tz C2–C7, Mean ADH C2–C7, and Mean PDH C2–C7). This resulted in six primary comparisons. To account for the possibility of inflated type I error due to multiple comparisons, we considered a conservative Bonferroni correction. Under this approach, the traditional alpha level of 0.05 is divided by the number of tests performed (0.05/6 = 0.003).

The post hoc power analyses were conducted based on the output for primary outcomes. The power was derived as 93.24% and 92.11% for ARA C2–C7 and Tz C2–C7, respectively, which meant that the sample size of 64 patients was sufficient for the statistical analysis.

## 4. Discussion

The results of this case series show that CBP^®^ is successful in correcting cervical spinal alignment and posture by using MI corrective spinal adjustments, therapeutic spinal exercises, and mechanical spinal traction. This case series shows a positive correlation (direct relationship) as unhealthy, abnormal cervical lordosis and anterior head translation are improved toward a healthy, normal (by decreasing in value), so too did NP and disability improve significantly (also by decreasing in value). Additionally, this case series shows a negative correlation (inverse relationship) as unhealthy, abnormal cervical lordosis and anterior head translation improved (by decreasing in value), so too did anterior and posterior cervical disc height (by increasing in value); and as anterior and posterior disc height improved (by increasing in value), so too did neck pain and disability (by decreasing in value).

### 4.1. Spinal Alignment and Posture

Abnormal, unhealthy cervical spinal alignment and posture have been shown to cause cervical spine degeneration. Loss or reversal in cervical lordosis [4,5,6,7,8,9], increased anterior head posture [10,11], and abnormal cervical biomechanics [38,39,40,41] are shown to have a causative effect. Loss of cervical lordosis and the progressive decline in cervical stability can be attributed to the accumulation of cervical acceleration–deceleration trauma (whiplash), long-term abnormal posture loading, poor ergonomic and lifestyle choices, and tissue damage in the neck [8]. Abnormal sagittal cervical spinal alignment, such as cervical hyper- or hypolordosis, cervical straightening, cervical kyphosis, and sagittal buckling of the cervical spine, results in abnormal cervical biomechanics, dysfunction, bone spur formation, damage to cervical musculature, and cervical spondylosis and DDD [8]. There exists a negative relationship between cervical spine degeneration and the extent of cervical lordosis in patients experiencing NP. Specifically, increased cervical disc herniation and spinal cord compression are associated with decreased lordosis [8].

With cervical disc degeneration, the body utilizes protective mechanisms, including increased spinal muscle tension, for the purposes of increasing stabilization [42]. However, this also results in decreased mobility, flexibility, and ROM, cervical spine dysfunction, and abnormal spinal biomechanics, creating a vicious cycle of reciprocal cause and effect in which these elements further intensify and exacerbate cervical DDD, which, in turn, further intensifies and exacerbates these elements, leading to a continuously worsening situation [42].

A healthy cervical lordosis is vital for preserving balance and motor function and serves as a primary factor for cervical disc degeneration because of its load-bearing role. In addition to dampening vibration and compression, a healthy cervical curvature is a crucial component of normal spinal biomechanics and maintains the stability of the spine [8].

Improvement in cervical lordosis redistributes mechanical loading across the intervertebral disc as seen in cervical extension. Biomechanically, this may lead to increased decompressive forces at the anterior aspect of the disc. Due to the heterogeneity in disc material properties and intradiscal pressures, it is reasonable to expect asymmetric changes. This mirrors observations in DDD in which asymmetric loading leads to asymmetry in joint and disc degeneration from anterior to posterior. These notions are confirmed by Wolff’s law of bone remodeling [43], Davis’ law of soft tissue adaptation [44], and Heuter–Volkmann law [45], which show how mechanical forces affect bony and soft tissue growth and adaptation.

The observed changes in cervical disc height may be the result of mechanical remodeling and the reduction of inflammation associated with disc degeneration. Restoration of cervical lordosis may alleviate the abnormal mechanical loading that has been shown to induce pro-inflammatory cytokine expression and neuroinflammatory responses at both the cellular and tissue levels [46,47,48]. Biomechanical studies have demonstrated that adverse mechanical loading alone, independent of chronological age, can precipitate adjacent segment degeneration [42]. The inflammatory response following abnormal loading, including increased expression of matrix-degrading enzymes and cytokines, has been well documented in both in vitro and animal models [36,46]. Restoring cervical lordosis may confer biomechanical and biochemical benefits by redistributing load, improving disc space, and minimizing inflammatory activation. These mechanisms are well-aligned with prior evidence linking mechanical injury to disc degeneration and pain generation.

There are those who deny that the physiological alignment of the cervical spine is a lordosis. They cite literature that supports their claims based on defined normative anatomical values for cervical spinal alignment, relying on data from normal, healthy subjects. The problem with these studies is the use of asymptomatic as a qualification for normal or healthy; asymptomatic is not interchangeable with normal or healthy. For example, studies have identified high prevalence of coronary plaque [49,50] and early cancers [51] in asymptomatic adult populations, accounting for the top two causes of mortality in the United States (US) and globally (cardiovascular disease and cancer) [52]. Approximately 40% of adults in the US are obese [53]; approximately 40% of adults in the US will be diagnosed with cancer [54]; approximately 50% of adults in the US have hypertension [55]. Patients with these conditions are not healthy based on the presence or absence of their symptoms. Their position on the health spectrum may differ based on the severity of symptoms or the condition, but the presence of these conditions negates the designation of healthy. As such, while there may be studies that show cervical kyphosis in asymptomatic subjects, this does not establish it as normal. This may identify the prevalence or incidence in a given population or time period, but this conclusion is making a serious error in conflating normal with common.

In a 2022 study, Charles et al. identified that “50.9% [of an asymptomatic population] presented an evenly distributed cervical lordosis. Kyphotic [cervical] alignment represented only 1.3%… Sigmoid patterns were also found in [the] asymptomatic population. Proximal kyphosis and distal lordosis represented 34.4%… Proximal lordosis and distal kyphosis represented 13.4% [56].” Also from the Charles et al. study, “Intervertebral disc degeneration, facet joint osteoarthritis and degenerative spondylolisthesis without thoracolumbar malalignment did not represent exclusion criteria since these radiographic changes commonly occur during aging.” First, these radiographic changes correlate with alignment and posture as opposed to aging.

In lordosis, anterior and posterior stresses in the vertebral body are nearly uniform and minimal. In kyphotic areas, combined stresses changed from tension to compression at the anterior vertebral margins and were very large (6–10 times as large in magnitude) compared to lordosis. In kyphotic areas at the posterior vertebral body, the combined stresses changed from compression (in lordosis) to tension. The stresses in kyphotic areas are very large and opposite in direction compared to a normal lordosis. This analysis provides the basis for the formation of osteophytes (Wolff’s Law) on the anterior margins of vertebrae in kyphotic regions of the sagittal cervical curve. This indicates that any kyphosis is an undesirable configuration in the cervical spine. Osteophytes and osteoarthritis are found in areas of altered stress and strain. Axial and flexural stresses at kyphotic areas in the sagittal cervical spine are abnormally high [57].

Second, the inclusion of spinal pathologies in this study eliminates the designation of the population as normal or healthy. These findings are similar to other studies that claim to report on normative data.

In a 2017 study by Kim et al., “as the participants were considered volunteers and not patients, there was no way to determine if they had sustained injuries or received treatments on their spines [58].” In a 2012 study by Yukawa et al., “a total of 1230 healthy Japanese volunteers were enrolled in this study. The exclusion criteria included a history of brain or spinal surgery, comorbid neurologic disease such as cerebral infarction or neuropathy, symptoms related to sensory or motor disorders (numbness, clumsiness, motor weakness, and gait disturbances) or having severe neck pain [59].” One glaring issue is that volunteers with moderate pain would be included in this study because it is less than severe. In a 2019 study, Le Huec et al. report on “a group of 25 patients with non-specific NP…and 25 age-, sex- and BMI-matched volunteers who had no history of NP at least for 1 year before their enrollment…as the control group [60].” One issue is that volunteers could have had a history of neck pain for up to 1 year. The inclusion of spinal pathologies and trauma eliminates the designation of these populations as normal or healthy.

So, how can healthy spinal alignment be determined? First, geometric modeling studies can identify theoretical ideal values [27]. Then, there are studies that evaluate observed radiographs against geometric modeling along a spinal health continuum, considering spinal conditions and symptoms and their severities with respect to spinal alignment [29,30]. Going further, there are studies that evaluate the effects of improvement and/or deterioration of cervical spinal alignment on spinal conditions and symptoms and their severities [61,62,63,64,65,66,67,68,69,70,71,72]. These reviews and clinical trials show that improvement in cervical lordosis results in long-term improvement in various spinal pathologies and comorbidities, while no changes are observed when control groups are included, and only immediate, short-term symptomatic improvements are documented when treatments do not affect cervical spinal alignment and posture. These studies show direct relationships between the severity of spinal alignment and posture and patient symptoms and function. In other words, worsening spinal alignment and posture are associated with worsening severity of symptoms and disabilities. The magnitude of spinal alignment and posture improvements correlates with the improvements in symptoms and function. These studies confirm or deny healthy spinal measures because they consider the participants as they are, instead of manipulating definitions of health to categorize them.

There is published literature reporting improvements in functional and symptomatic health outcomes in patients with cervical DDD following conservative spinal therapies. However, there do not appear to be any studies that investigate the impact of improving cervical spinal alignment and posture on cervical DDD. This case series may be the first research evidence to report on improved functional, symptomatic, and radiographic measures of cervical DDD in 64 patients following structural cervical spine rehabilitation per CBP protocols.

### 4.2. Limitations

This case series provides a clear objective and well-defined protocol. Additionally, this case series includes objective and subjective clinical outcomes with valid and reliable quantified measurements. This helps to strengthen the value of the series. This case series is retrospective with strict inclusion criteria, which limit its generalizability to larger populations of patients. However, this case series is also consecutive, which means that all patients who met the inclusion criteria within a designated time range were included in the study, and those who met the exclusion criteria were excluded, which reduces selection bias. This series reports on subjects with a wide age range (23–77 years), which can include different degrees of degeneration, which limits its application to stratified populations. Stratified analyses would not have been appropriately powered. Post hoc power analysis should be interpreted with caution, as it relies on observed data rather than pre-specified assumptions. This study would have benefited from a control group, magnetic resonance imaging (MRI) of the cervical spine to assess Modic changes, soft tissue damage, as well as spinal cord compression and canal stenosis, and three dimensional (3-D) advanced imaging (e.g., 3-D computed tomography (CT) or MRI) as a gold standard for more precise morphological measurements of the disc heights to bolster the validity of these findings. Future studies would benefit from a prospective design comparing multiple groups (including a control) with stratified age ranges and degrees of degeneration and using a range of advanced imaging (e.g., radiography and MRI or CT) for bony and soft tissue assessment (e.g., the Pfirrman grading scale, Thompson system for disc degeneration, etc.). This study does not show causation, has no control group, and limits generalizability to apply to other populations. Larger prospective clinical trials with α priori power analysis, control groups, standardization (e.g., consistent number of treatment sessions, treatment duration, etc.), and long-term follow-ups are needed to verify these outcomes in patients with cervical DDD and CBP^®^ structural spinal rehabilitation. Long-term follow-ups will help to verify whether any changes in disc height are structural recovery of the discs. Future studies should include coronal cervical radiographs, oblique cervical radiographs, flexion and extension cervical radiographs, pre- and post-treatment MRI, and more measurements (e.g., sagittal and coronal balance, cervical and thoracic morphology, etc.) to further assess spondylosis, degeneration, and biomechanics, alignment, and stability of the cervical spine.

## 5. Conclusions

Currently, cervical DDD is a wait-and-watch condition with no restorative treatment guidelines. CBP^®^ focuses on restoring healthy alignment and biomechanics of the spine and posture [27]. This case series shows that CBP^®^ spinal rehabilitation may be an effective conservative, non-surgical treatment for cervical degeneration patients with abnormal cervical spinal alignment and posture, neck pain, and disability. By using CBP^®^ spinal rehabilitation to improve spinal alignment and postural distortions, the need for medical or invasive surgical procedures may be negated. Future prospective studies involving larger populations, multiple clinic sites, controlled and experimental groups, and long-term follow-ups, will shed more light on the effectiveness and reliability of CBP^®^ in correcting cervical spinal alignment and posture and the associated functional and symptomatic effects and pathologies.

## Figures and Tables

**Table 1:** Patient demographics and treatment information.

Patient Information	
Patients (n)	64
Males	19 (29.7%)
Females	45 (70.3%)
Mean Height (cm)	171.93 ± 1.85
Mean Weight (kg)	68.12 ± 2.53
Mean Age (y)	49.05 ± 3.34
Mean Treatment Visits (n)	37.80 ± 2.44
Mean Duration of Treatment (wk)	19.53 ± 3.89

n = number, cm = centimeter, kg = kilogram, y = year, wk = week.

**Table 2:** Comparison of pre-treatment and post-treatment patient outcome assessments.

Outcome Assessment	Pre-CBP Treatment (95% CI)	Post-CBP Treatment (95% CI)	Mean Difference (95% CI)	*p*-Value
ARA C2–C7 (°)	−6.18 ± 3.06	−19.95 ± 3.05	13.77 ± 8.80	*<0.0001*
Tz C2–C7 (mm)	22.03 ± 2.39	12.11 ± 2.34	9.91 ± 5.77	*<0.0001*
Anterior Disc Height C2–C7 (mm)	3.68 ± 0.20	5.19 ± 0.21	1.52 ± 0.59	*<0.0001*
ADH C2–C3 (mm)	3.96 ± 0.21	5.40 ± 0.27	1.45 ± 0.96	*<0.0001*
ADH C3–C4 (mm)	4.04 ± 0.25	5.39 ± 0.27	1.35 ± 0.98	*<0.0001*
ADH C4–C5 (mm)	3.70 ± 0.31	5.23 ± 0.28	1.53 ± 0.91	*<0.0001*
ADH C5–C6 (mm)	3.33 ± 0.28	4.87 ± 0.32	1.54 ± 0.88	*<0.0001*
ADH C6–C7 (mm) **	3.36 ± 0.29	5.08 ± 0.31	1.72 ± 0.93	*<0.0001*
Posterior Disc Height C2–C7 (mm)	3.22 ± 0.15	4.35 ± 0.16	1.14 ± 0.50	*<0.0001*
PDH C2–C3 (mm)	3.60 ± 0.23	4.81 ± 0.26	1.21 ± 0.99	*<0.0001*
PDH C3–C4 (mm)	3.49 ± 0.20	4.49 ± 0.24	1.01 ± 0.87	*<0.0001*
PDH C4–C5 (mm)	3.28 ± 0.23	4.31 ± 0.21	1.03 ± 0.93	*<0.0001*
PDH C5–C6 (mm)	2.86 ± 0.21	3.99 ± 0.20	1.13 ± 0.71	*<0.0001*
PDH C6–C7 (mm) **	2.83 ± 0.23	4.19 ± 0.23	1.36 ± 0.78	*<0.0001*
Neck Pain Numeric Rating Scale	6.66 ± 0.27	1.52 ± 0.26	5.14 ± 0.56	*<0.0001*
Neck Disability Index (%)	40.28 ± 1.42	12.66 ± 0.96	27.63 ± 4.46	*<0.0001*

CBP = Chiropractic BioPhysics, CI = confidence interval, ARA = absolute rotational angle of measurement, ° = degree, Tz = translation in the *z*-axis, mm = millimeter, ADH = anterior disc height, PDH = posterior disc height, ** = the largest change in intervertebral pre- to post-disc height measurements, significant *p* values are italicized.

**Table 3:** Multivariable analysis for primary outcomes through generalized linear model.

Parameters	MODEL I: Outcome = ARA C2–C7		MODEL I: Outcome = Tz C2–C7	
	Estimate (95% CI)	*p*-Value	Estimate (95% CI)	*p*-Value
Treatment: Post vs. Pre	37.61 [29.1–46.11]	*<0.0001*	6.52 [−3.1–16.15]	0.1838
Metric				
Anterior Disc Height C2–C7 (mm)	−2.75 [−5.97–0.48]	0.0950	1.09 [−2.56–4.73]	0.5593
Posterior Disc Height C2–C7 (mm)	1.81 [−2.35–5.98]	0.3940	−3.03 [−7.74–1.68]	0.2071
Neck Pain Numeric Rating Scale	5.36 [3.04–7.68]	*<0.0001*	3.16 [0.54–5.78]	*0.0183*
Neck Disability Index (%)	0.79 [0.29–1.28]	*0.0019*	−0.06 [−0.62–0.5]	0.8370

ARA C2–C7 = absolute rotational angle from C2 to C7, Tz C2–C7 = translation in the *z*-axis of C2 with respect to C7, CI = confidence interval, mm = millimeter, % = percentage, significant *p* values are italicized.

## Data Availability

The data for this study are included in the article. Further inquiries can be directed to the corresponding author.

## Abbreviations

The following abbreviations are used in this manuscript:

NP	neck pain
YLD	years lived with disability
ROM	range of motion
HA	headache
DDD	degenerative disc disease
CBP^®^	Chiropractic BioPhysics^®^
ICD-10	International Classification of Diseases, Tenth Revision
PT	physical therapy
PRO	patient-reported outcome
NRS	numeric rating scale
NDI	neck disability index
MI	Mirror Image^®^
NLC	neutral lateral cervical
C2-C7	second through seventh cervical vertebrae
IRB	institutional review board
PROM	patient-reported outcome measure
MCID	minimal clinically important difference
CI	confidence interval
°	degree
mm	millimeter
ADL	activities of daily living
HPTM	Harrison posterior tangent method
ARA	absolute rotation angle
ADH	anterior disc height
PDH	posterior disc height
MAD	mean absolute deviation
T,R	Translation, Rotation
x,y,z	x-, y-, z-axes
H,T,P	Head, Thorax, Pelvis
EHR	electronic health records
P-A	posterior-to-anterior
CNS	central nervous system
DV	dependent variables
IV_i_	independent variables
ε_i_	the idiosyncratic error term
β_i_	average effect
MRI	magnetic resonance imaging
CT	computed tomography
US	United States
